# Breast Cancer Genetics: Diagnostics and Treatment

**DOI:** 10.3390/genes13091593

**Published:** 2022-09-06

**Authors:** Carmen Criscitiello, Chiara Corti

**Affiliations:** 1 Division of New Drugs and Early Drug Development for Innovative Therapies, European Institute of Oncology, IRCCS, 20141 Milan, Italy; 2 Department of Oncology and Haematology (DIPO), University of Milan, 20122 Milan, Italy

Breast cancer (BC) genetics has become a fundamental aspect of BC management.

It influences screening, follow-up, prophylactic and therapeutic recommendations in women harboring a germinal BC susceptibility gene. In addition, it helps to identify patient subgroups with either a different prognosis or different response to treatment. 

This Special Issue consists of one case report, two original research articles and five reviews, covering both diagnostic aspects and therapeutic implications of genetics in BC.

Pathogenic variants in the BC susceptibility genes represent the strongest hereditary risk factor for disease development, particularly in the context of early onset breast cancer (EOBC). Indeed, around 10–20% of EOBC cases are hereditary [1]. Consequently, individuals with a personal or family history of breast, ovarian, prostate or pancreatic cancer may benefit from hereditary risk evaluation to determine their own risk and family members’ risk for these and associated cancers. In this regard, Szczerba and colleagues examined 75 tumor samples from a cohort of Polish BC patients that had negative results for targeted breast cancer susceptibility genes 1 (*BRCA1*) mutations (c.5266dupC, c.181T > G, c.4035delA, c.68_69delAG, c.3700_3704delGTAAA). All coding regions of the *BRCA1/2* genes were sequenced with Next Generation Sequencing (NGS), with the detection of nine pathogenic variants and six variants of unknown significance (VUS). The authors also focused on methodological aspects of NGS, highlighting differences in variant calling files (VCF) obtained from the same FASTQ file, according to the variant calling algorithm used. The authors conclude that this observation could potentially affect the identification and interpretation of variants [2].

Moreover, recent studies have also shown germline *BRCA1/2* status to be clinically relevant in the selection of therapy for patients already diagnosed with BC. Indeed, *BRCA* status predicts responsiveness to platinum-based chemotherapy as well as to inhibitors of poly(ADP-ribose) polymerase (PARP), highlighting the ability of these interventions to inhibit DNA repair pathways. From a surgical standpoint, surgical risk reduction remains a powerful tool in the therapeutic armamentarium for many women with genetic predisposing variants, as comprehensively highlighted by Berger and Golshan [3]. However, initial BC and contralateral BC risks should be clearly identified (i.e., highly penetrant genes compared to moderately penetrant genes), in order to fine tune risk reduction strategies and ideal timing, also in accordance with patient’s personal preferences [3]. While the survival benefit related to prophylactic bilateral mastectomy has been established, a growing body of evidence supports the oncological safety of nipple-sparing mastectomy as a risk-reducing procedure in *BRCA*-mutated patients, with low rates of new BCs, low rates of postoperative complications and high levels of satisfaction and postoperative quality of life, as reported by Rocco et al. [4]. However, larger multi-institutional studies with longer follow-up are needed to establish this procedure as the best surgical option in this setting.

Besides *BRCA1/2*, pathogenic variants in other high- to moderate-risk genes such as tumor protein p53 (*TP53*), partner and localizer of BRCA2 (*PALB2*), phosphatase and tensin homolog (*PTEN*), checkpoint kinase 2 (*CHEK2*) and ataxia-telangiectasia mutated (*ATM*) account for a smaller percentage of BC, and, in some cases, ovarian, prostate or pancreatic cancers [5,6,7,8]. 

In particular, ATM is involved in cell cycle control, apoptosis, oxidative stress and telomere maintenance, and its role as a risk factor for cancer development is well established [9]. Recent studies confirmed that some variants of *ATM* are associated with intermediate- and high-grade disease, a higher rate of lymph node metastatic involvement, HER2 positivity as well as the development of a contralateral breast tumor, as depicted by Stucci and colleagues [9]. Clinicopathologic characteristics of BC developed by *ATM* and checkpoint kinase 2 (*CHEK2*) mutation were also explored by Toss and colleagues, who reviewed the archive of the local Family Cancer Clinic. Since 2018, 1185 multi-gene panel tests were performed. In total, 19 *ATM* and 17 *CHEK2* mutation carriers affected by 46 different BCs were identified. A high rate of bilateral tumors was observed in *ATM* (26.3%) and *CHEK2* mutation carriers (41.2%). While 64.3% of *CHEK2*-mutant tumors were luminal A-like, 56.2% of *ATM*-mutant tumors were luminal B-like/HER2-negative. Moreover, 21.4% of *CHEK2*-related invasive tumors showed a lobular histotype. About a quarter of all *ATM*-related BCs and a third of *CHEK2*-related BCs were *in situ* carcinomas and more than half of *ATM*- and *CHEK2*-related BCs were diagnosed at stage I-II. The biological and clinical characteristics of *ATM*- and *CHEK2*-related tumors may help improve diagnosis, prognostication and targeted therapeutic approaches. Importantly, the authors advise the consideration and discussion of contralateral mastectomy for *ATM* and *CHEK2* mutation carriers at the first diagnosis of BC.

This growing body of data regarding the identification of new *ATM* aberration as well as association with ancestry, prognosis and treatment outcomes could support clinicians in personalizing both treatments, as well as follow-up, in these patients [10]. Moreover, since mutations in *ATM* are involved in DNA repair mechanisms, ATM aberrations may sensitize cancer cells to platinum-derived drugs and PARPi, as *BRCA1/2* mutations do. Some evidence suggests that *ATM* mutations could also be involved in the resistance to cyclin-dependent kinase 4 and 6 inhibitors (CDK4/6i) in luminal BC [10]. 

In this context, publicly available archives and case reports highlighting relationships among human gene variants and phenotypes are of particular importance. For example, Parenti et al. identified a new *ATM* deletion associated with a *BRCA*-negative patient who developed BC at the age of 34 [10]. Her mother had unilateral receptor-positive BC at the age of 45 with axillary lymph node involvement. The authors utilized SOPHiA Genetics Hereditary Cancer Solutions gene panel to detect a copy number variant (CNV), that was first validated by Multiplex Ligation-dependent Probe Amplification (MLPA). Afterward, long-range Polymerase Chain Reaction (PCR) and Sanger sequencing were used to characterize the breakpoint at DNA level (c.2838+2162_4110-292del) in proband and to also study segregation in the patient’s mother and sister. Further characterization at the RNA level on the proband’s mother and sister identified the presence of both the wild-type and the mutant allele in the mother’s sample. This abnormal ATM protein lacks the domain required for c-Abl protein interaction and mediation of cell cycle arrest in G1 phase. In addition, at least three other important domains are deleted from the ATM protein, such as the FAT (FRAP-ATM-TRRAP), PIKK (phosphatidylinositol 3-kinase-related kinase domain) and FATC (FAT C-terminal domain) domains, mediating most ATM functions.

Siddig et al. focused more broadly on the genetic landscape of EOBC, since 10–20% of these cancers are related to germline BC susceptibility genes. The authors provide an overview of somatic mutations, chromosome CNVs, single-nucleotide polymorphisms (SNPs), differential gene expression, microRNAs and gene methylation profile as well as of altered pathways resulting from those aberrations. Interestingly, the E-Cadherin/β-Catenin complex and the overall determinants of epithelial barrier integrity have been implicated in EOBC, with cell–cell adhesion genes such as *CDH1*, *GATA3*, *CTNNB1*, *MUC17* and *FLG* involved [1,11]. Eight stromal genes are differentially expressed in breast tumors from very young patients (≤35 years) compared to tumors from older age patients (≥50 years), with *UQCRQ*, *ALDH1A3*, *EGLN1* and *IGF1* being overexpressed and *FUT9*, *IDI2*, *PDHX* and *CCL18* being underexpressed. The *TP53* gene typically shows a high mutational load in EOBC and plays an important pathogenic role by affecting cell cycle arrest mechanisms and the transcription of other genes, such as *GAS7b*, which regulates the cell structure and cell migration [12]. EOBC aggressive characteristics also appear to be linked to DNA methylations events [13]. EOBC displays several CNVs implicated in tumorigenesis (6q27, 6p32 and 7p21.1), advance-stage tumor progression (22q12.3 and 22q13.31), disease progression (19q13.32) and prognosis (CNV in *BIRC5* gene). However, further studies that correlate the CNV profile with the gene and protein expression profile are needed. Finally, different SNPs may be linked to EOBC tumorigenesis, progression, resistance to chemotherapy and poor prognosis. Additionally, it is possible to discriminate BC arising in young women from that in older women using a microRNA profile [1]. 

In terms of future perspectives, even though several disease-causing mutations have been identified, therapy is often aimed at interfering with an aberrantly activated pathway, rather than rectifying the mutation in the DNA sequence. The Clustered Regularly Interspaced Short Palindromic Repeats (CRISPR)/Cas9 is a groundbreaking tool that is being utilized for the identification and validation of genomic targets bearing tumorigenic potential. CRISPR/Cas9 supersedes its gene editing predecessors through its unparalleled simplicity, efficiency and affordability. Ahmed and colleagues provide an overview of the CRISPR/Cas9 mechanism and discuss genes that were edited using this system for the treatment of BC. In addition, the authors shed light on the delivery methods, both viral and non-viral, that may be used to deliver the system, as well as on the main challenges associated with each method. However, despite great expectations, remarkable limitations related to ethics, off-target effects, mutagenesis and delivery necessitate further studies. For the conventional use of this system in the near future, both precise knowledge of pathogenic variants as well as the optimization of the system itself are essential.

In conclusion, the papers in this Special Issue cover various aspects of genetics in BC. Overall, they provide a summary of hereditary BC syndromes, personalized BC risk assessments, as well as historical and novel risk reduction approaches. They also offer a comprehensive overview regarding major advances in understanding the most frequent genetic aberrations, with potential implications for present and future treatment approaches.
